# Attenuation of Immune-Mediated Influenza Pneumonia by Targeting the Inducible Co-Stimulator (ICOS) Molecule on T Cells

**DOI:** 10.1371/journal.pone.0100970

**Published:** 2014-07-16

**Authors:** Priya Sakthivel, Marcus Gereke, Angele Breithaupt, Dietmar Fuchs, Luca Gigliotti, Achim D. Gruber, Umberto Dianzani, Dunja Bruder

**Affiliations:** 1 Immune Regulation Group, Helmholtz Centre for Infection Research, Braunschweig, Germany; 2 Infection Immunology Group, Institute of Medical Microbiology, Infection Control and Prevention, Otto-von-Guericke University, Magdeburg, Germany; 3 Department of Veterinary Medicine, Institute of Veterinary Pathology, Free University, Berlin, Germany; 4 Division of Biological Chemistry, Biocenter, Innsbruck Medical University, Innsbruck, Austria; 5 Department of Health Sciences and Interdisciplinary Research Center of Autoimmune Diseases, “A. Avogadro” University of Eastern Piedmont, Novara, Italy; University of Duisburg-Essen, Germany

## Abstract

Inducible Co-stimulator (ICOS) plays a critical role in mediating T cell differentiation and function and is considered a key player in balancing T effector and T regulatory (T_reg_) cell responses. Here we show that activation of the ICOS signalling pathway during acute influenza A virus (IAV) infection by application of an agonistic ICOS antibody reduced the frequency of CD8^+^ T cells in the respiratory tract of IAV infected animals and delayed pathogen elimination. In line with this, immune-mediated influenza pneumonia was significantly ameliorated in mice that received ICOS agonist as indicated by significantly reduced alveolar infiltrations and bronchointerstitial pneumonia, while at the same time virus-related pathology remained unaffected. Importantly, ICOS agonist treatment resulted in expansion of CD4^+^Foxp3^+^ T_regs_ in IAV infected mice, which was associated with elevated levels of the immunosuppressive cytokine IL-10 in the alveolar space. Together, our findings suggest a prominent role of ICOS signaling during acute IAV infection by increasing the T_reg_/CD8^+^ T cell ratio with beneficial outcome on immune-mediated pneumonia and underline the suitability of ICOS as potential therapeutic target for immune intervention in those infectious conditions characterized by strong immunopathology rather than virus-mediated cytopathic effects.

## Introduction

Inducible co-stimulator (ICOS) is a T cell co-stimulatory molecule that belongs to the CD28 Immunoglobulin (Ig) superfamily [1]. It is primarily expressed on activated T cells and mediates its immunological functions by binding to its ligand ICOS-L [2], [3]. ICOS/ICOS-L interaction provides a co-stimulatory signal to the T cells resulting in the amplification of T cell activation and enhanced proliferation as well as cytokine secretion [4]. ICOS signaling was shown to play an essential role for the differentiation of T cells into Th1 and Th2 cells following antigen-specific activation [5]–[8] and a recent study emphasizes that ICOS plays an important role also in promoting Th17 responses [9]–[11]. Germinal center formation is impaired in mice lacking ICOS and ICOS plays a critical role in co-stimulating humoral immune responses [12], [13]. So far, ICOS-mediated co-stimulation has been predominantly reported to play a role in CD4^+^ T helper cell subsets, but there is accumulating evidence for an effect of ICOS also on CD8^+^ T cells [14]–[16].

Next to its multifaceted role in the initiation of adaptive immune responses, ICOS has also been described to be crucially involved in the regulation of adaptive immunity. While ICOS was originally shown to enhance T cell proliferation, differentiation and induction of certain effector cytokines [17], ICOS/ICOSL interaction was more recently shown to specifically super-induce the synthesis of the anti-inflammatory cytokine IL-10 [18], [19]. Interestingly, thymus-derived regulatory T cells (T_reg_) can be subdivided according to the expression of ICOS, with ICOS^+^FOXP3^+^ T_reg_ releasing both, IL-10 to suppress dendritic cell function and TGF-β to suppress T cell function, in contrast to ICOS^−^Foxp3^+^ T_reg_ secreting TGF-β only [20], [21]. Blockade or absence of ICOS inhibited the production of IL-10 and abrogated the inhibitory function of T_regs_ [22], [23]. As demonstrated in a murine model for autoimmune diabetes, disturbance of the balance between T effector (T_eff_) and T_reg_ cells by interference with the ICOS signaling pathway led to the conversion of early insulitis to diabetes indicating that T_reg_ prevented pancreatic islet destruction in an ICOS-dependent mechanism [24]. In line with this, a defect in the induction of Foxp3 and ICOS expression was observed in newly diagnosed type 1 diabetic children [25]. Of note, a crucial role of ICOS has also been reported for the generation and suppressive function of T_reg_ conveying respiratory tolerance in a mouse model of allergic asthma [26]. Taken together, these existing reports clearly indicate that ICOS co-stimulates distinct effector and regulatory functions in T cells, thereby critically affecting the outcome of adaptive immune responses. The proposed function of ICOS to bias T cell responses *in vivo* makes this molecule a promising target for therapeutic intervention in numerous inflammatory and infectious conditions.

Studies on the specific role of ICOS in immunity to bacterial, viral or parasite infection have been greatly facilitated by the availability of mice lacking ICOS or using systemic delivery of blocking antibodies and revealed that the absence or blockade of ICOS leads to either unaffected of reduced CD4^+^ or CD8^+^ T cell responses [5], [14], [27]–[30]. For example, during *Mycobacterium tuberculosis* (Mtb) infection, it has been demonstrated that ICOS deficiency differentially affects CD4^+^ and CD8^+^ T cell subsets, ultimately resulting in improved protection in the spleen but not the lungs during later stages of Mtb infection [31]. While in this study ICOS deficiency resulted in a reduced Mtb-specific CD8^+^ T cell response, ICOS^−/−^ mice displayed no defect in the initial pathogen-specific CD8^+^ T cell expansion or cytotoxic effector function following influenza A virus (IAV) infection. However, ICOS was important in maintaining CD8^+^ T cell numbers in the late phase of the primary response [32]. In another study, on the role of ICOS co-stimulation for the outcome of IAV infection that utilized antibody-mediated ICOS inhibition, a marked reduction of cytokine-secreting CD4^+^ and CD8^+^ T cells was observed in the lung. This was associated with reduced pulmonary T cell inflammation and elevated lung viral titers but at the same time increased soluble inflammatory mediators were detected in the lung [30]. Together, these examples suggest that selective blockade of ICOS signaling biases T cell responses *in vivo* with implications for the course of infection.

To our knowledge, *in vivo* studies on the potential role of ICOS in T cell activation, differentiation and function have so far been performed in either ICOS^−/−^ mice or by antibody-mediated ICOS blockade. In contrast to this, in the present study we combined IAV infection with systemic delivery of a stimulating anti-ICOS antibody to super-induce ICOS signaling in T cells. Our studies revealed that ICOS agonist treatment during acute IAV infection resulted in reduced CD8^+^ T cell levels in the respiratory tract while at the same time the frequency of peripheral CD4^+^Foxp3^+^ T_regs_ was increased. Changes in the T_reg_ to CD8^+^ T cell ratio were associated with delayed viral clearance, increased IL-10 concentration in the bronchoalveolar space and a significantly attenuated immunopathology in the lung of IAV infected mice. Together, these data suggest that ICOS agonist treatment during acute IAV infection has a beneficial effect on immune-mediated influenza pneumonia, most likely caused by an increased T_reg_/CD8^+^ T cell ratio, supporting the idea that ICOS can be considered a promising molecular target for future development of specific *in vivo* immune intervention strategies.

## Materials and Methods

### Mice, ICOS antibodies and experimental procedure

All animal experiments were approved by the Niedersächsisches Landesamt für Verbraucherschutz und Lebensmittelsicherheit. Eight to ten week old female BALB/c mice were obtained from Harlan Winkelmann (Rossdorf, Germany) and were used for this study. Mice were maintained under specific pathogen-free conditions in the animal facility at the Helmholtz Centre for Infection Research, Braunschweig, Germany. For sub-lethal intranasal influenza infection mice were anaesthetized by Ketamine/Rompun (dose of 0.1 ml/10 g of mice) and a volume of 25 µl of the PR/8/34 (H1N1) virus strain containing the appropriate concentration (a dose lethal to 50% of inoculated BALB/c mice (MLD50)) of virus was administered onto the nostrils. Following infection, mice were divided into two experimental groups: the first group received 200 µg of anti-ICOS agonist antibody on day 1 and 100 µg ICOS agonist on day 5 post-infection, whereas the control group received the same doses of hamster IgG isotype control antibody. Alternatively, in one experimental setting, two additional groups were included that received anti-ICOS antagonist antibodies and rat IgG isotype controls, respectively. Mice were sacrificed and analyzed during the peak phase of adaptive immunity (day 7–9), as indicated in the figure legends. All experiments were performed in accordance to the institutional and national guidelines. Anti-ICOS agonist antibody (clone C398.4A) was produced and purified as described before [33]. Of note, in order to exclude the possibility that potential effects of *in vivo* anti-ICOS agonist treatment might be a result from blocking ICOS-ICOS-L interaction rather than from agonistic signaling following ICOS agonist binding to T cells, activated splenic T cells were pre-incubated with ICOSL-Fc followed by C398.4A staining. Subsequent FACS analysis revealed only minor blockade of C398.4A binding by soluble ICOS-L pre-incubation (**Figure S1**). In addition, we could clearly rule out the possibility that *in vivo* ICOS agonist treatment results in depletion of ICOS^+^ T cells (**Figure S2**). Polyclonal hamster IgG isotype control antibody, anti-ICOS antagonist antibody (clone 17 G9) and the rat IgG_2_b isotype control antibody were purchased from Bio-X-cell, USA.

### Lymphocyte isolation and serum preparation

Mice were sacrificed and lungs, bronchial lymph nodes (BLN) and spleens were excised in order to obtain single cell suspensions, for further usage. Lungs were perfused prior to excision and lymphocytes were isolated by enzymatic tissue digestion and density gradient centrifugation as described before [34]. Cells from BLN were prepared by smashing them through 70 µm cell-strainers. Single cell suspensions were then washed and re-suspended in FACS buffer (phosphate buffer saline (PBS), 2% fetal calf serums (FCS), 0.5 M EDTA). Splenocytes were processed by flushing the spleen with ACK (ammonium chloride potassium) red cell lysis buffer and passing the cells through 70 µm cell strainers. Intermittent washing and centrifugation (1200 rpm) was performed at 4°C. The obtained single cell suspensions were re-suspended in FACS buffer. Cells from the bronchoalveolar space were isolated by flushing the lungs twice with 1 ml of PBS. Cells were then pooled and centrifuged at 2000 rpm for 10 minutes. In case lavage fluid was collected for further analysis, centrifugation of the collected supernatants was increased to 10,000 rpm for 10 minutes and stored at −70°C until usage. Cardiac puncture was done to collect blood for serum preparation. The blood samples were incubated at 37°C for 30 minutes and then cooled to 4°C for 30 minutes. These samples were then centrifuged at 2000 rpm for 10 minutes and sera were collected and stored at −20°C until usage.

### Flow cytometry

Single cell suspensions prepared from lungs, BLNs, spleens and bronchoalveolar lavage were stained with the indicated combinations of Alexa Fluor 780-conjugated anti-CD4 (RM4–5, eBioscience), anti-CD8-PEcy7 (53-6.7, BioLegend), biotinylated anti-ICOS (7E.17G9, BioLegend), biotinylated anti-CD43 (1B11, BioLegend) and APC/cy7streptavidin (BioLegend). For intracellular cytokine staining, cells were stimulated for 2 hours with phorbol 12-myristate 13-acetate (PMA) (0.1 µg/ml, Cat#p1585, Sigma) and ionomycin (1 µg/ml, Cat#I0634, Sigma) and with golgi stop (Brefeldin) (5 µl/ml, Cat#B7651, Sigma) for 2 hours, followed by permeabilization with NP-40 (igepal CA-630, Sigma) and staining with anti-IFNγ-APC (XMG1.2, BioLegend). Intracellular FoxP3 expression was determined using the FoxP3 staining buffer kit (FJK-16s, eBioscience) according to the manufacturer’s recommendations. For apoptosis assay, Annexin/7AAD staining was performed as described previously [35]. Flow cytometric analysis was performed using a FACSCanto or LSRFortessa (BD Biosciences) running with THE FACSDiva software (version 6.1.3) (BD Biosciences). Analysis was performed using FlowJo version 9.5.3 (Tree star, Inc.).

### *In vivo* cytotoxic T lymphocyte (CTL) assay

Target cells (splenocytes) loaded with virus-antigen were adoptively transferred into IAV infected mice on day 9 following infection. For each recipient, 2×10^7^ splenocytes were loaded with IAV nucleoprotein (NP) peptide antigen (TYQRTRALV), HZI Peptide Synthesis Platform and 2×10^7^ cells served as control. Antigen-loaded cells were stained with 2.5 µM carboxyfluoresceine succinimidyl ester (CFSE, Molecular Probes) (CFSE^high^) while unloaded control cells were stained with 0.25 µM CFSE (CFSE^low^). Cells were pooled and intravenously injected into recipients. These were sacrificed 4 hrs after transfer and the splenocytes were isolated and analysed by flow-cytometry. CFSE-positive lymphocytes were divided into CFSE^low^ and CFSE^high^ populations and percent lysis of target cells was calculated (r = (% CFSE^low^/% CFSE^high^); % lysis = (1–(r_uninfected_/r_infected_)×100).

### RNA isolation and Real time PCR

Lungs were homogenized in TriFastFL reagent (PeqLab). RNA was extracted by addition of 1-Br-3-Cl-Propane (Merck), purified by precipitation and treated with DNase (Ambion) prior to reverse transcription to cDNA (M-MLV Reverse Transcriptase, Invitrogen). RNA concentration was determined using a Nano Drop (ND-1000 spectrometer). Concentration of 2.5 ng of RNA was utilized for reverse transcription into cDNA. cDNA concentration was adjusted to 50 ng/µl for virus quantification using real-time PCR with primers specific for the viral nucleoprotein (NP) (5′-GAG GGG TGA GAA TGG ACG AAA AAC and 3′-CAG GCA GGC AGG CAG GAC TT) and a reference plasmid carrying the NP-sequence (pVI-PmH5-PR8-NP; pro­vided by G. Sutter, Munich, Germany) to obtain a CT-value/NP copy number standard. This standard was used to quantify NP copy numbers in the samples. All PCR analyses were performed in triplicates.

### Enzyme Linked Immuno-Sorbent Assay (ELISA)

BALF samples were collected by flushing the lungs with PBS followed by centrifugation as described above and were stored at −70°C until usage. IL-6, IFN-γ, TNF-α, and IL-10 concentrations in BALF were quantified by standard ELISA (BioLegend) according to the manufacturers instruction. All analyses were run in duplicates.

### High Performance Liquid Chromotography (HPLC)

Tryptophan and kynurenine concentrations in serum and BALF samples were determined by reversed-phase HPLC as described earlier [36]. Specimens were deproteinized with trichloroacetic acid and were separated on reversed phase C18 material using 0.015 mol/l potassium phosphate buffers (pH 6.4). Tryptophan was monitored by means of its native fluorescence (Varian ProStar 360, Palo Alto, CA) at 285 nm excitation and 360 nm emission wavelengths. Kynurenine was detected by ultraviolet absorption (Shimadzu SPD-6A, Korneuburg, Austria) at 365 nm wavelength in the same chromatographic run. Finally, kynurenine/tryptophan (kyn/trp) was calculated as an estimate of IDO activity [37].

### Histology

Whole lung sample were fixed in neutrally buffered 4% formaldehyde. Cross sections of the lungs were made with approximate 2 mm thickness. Tissue samples were removed from the fixatives and routinely paraffin embedded. Tissue sections (2 µm) of the paraffin-embedded tissue samples were cut and stained with hematoxylin and eosin (HE).

### *In vitro* Apoptosis Assay

Splenocytes from BALB/c mice were processed and used for this assay. The 96 well plate was coated with anti-CD3 antibodies (0.75 µg/ml) and incubated at 37°C for one hour. The plate was then washed twice with PBS. Equal numbers of splenocytes (5×10^5^ cells/well) were added along with different concentration of anti-ICOS agonist antibodies (0.01, 0.1, 1, 5 µg/well) and incubated (at 37°C) for 3 days. Splenocytes without anti-ICOS antibodies acted as activated control and splenocytes without any antibodies acted as unactivated control. All the samples were run in triplicates. The results were obtained with FACS acquisition as described [35].

### Statistics

Statistics were performed applying non-parametric Mann-Whitney test or unbiased unpaired t test as indicated in the figure legends using Graph Pad Prism 5.02 (Graph Pad Software, La Jolla). For the statistical analysis of IDO enzymatic activity, the Statistical Package for the Social Sciences (SPSS), Predictive Analytics Software Statistics (PASW Statistics 18, Chicago, IL, USA) was used. A p-value of <0.05 was considered significant (*p≤0.05; **p≤0.01; ***p≤0.001).

## Results

### ICOS agonist treatment does not affect the overall course of influenza infection in mice

**Figure 1:**
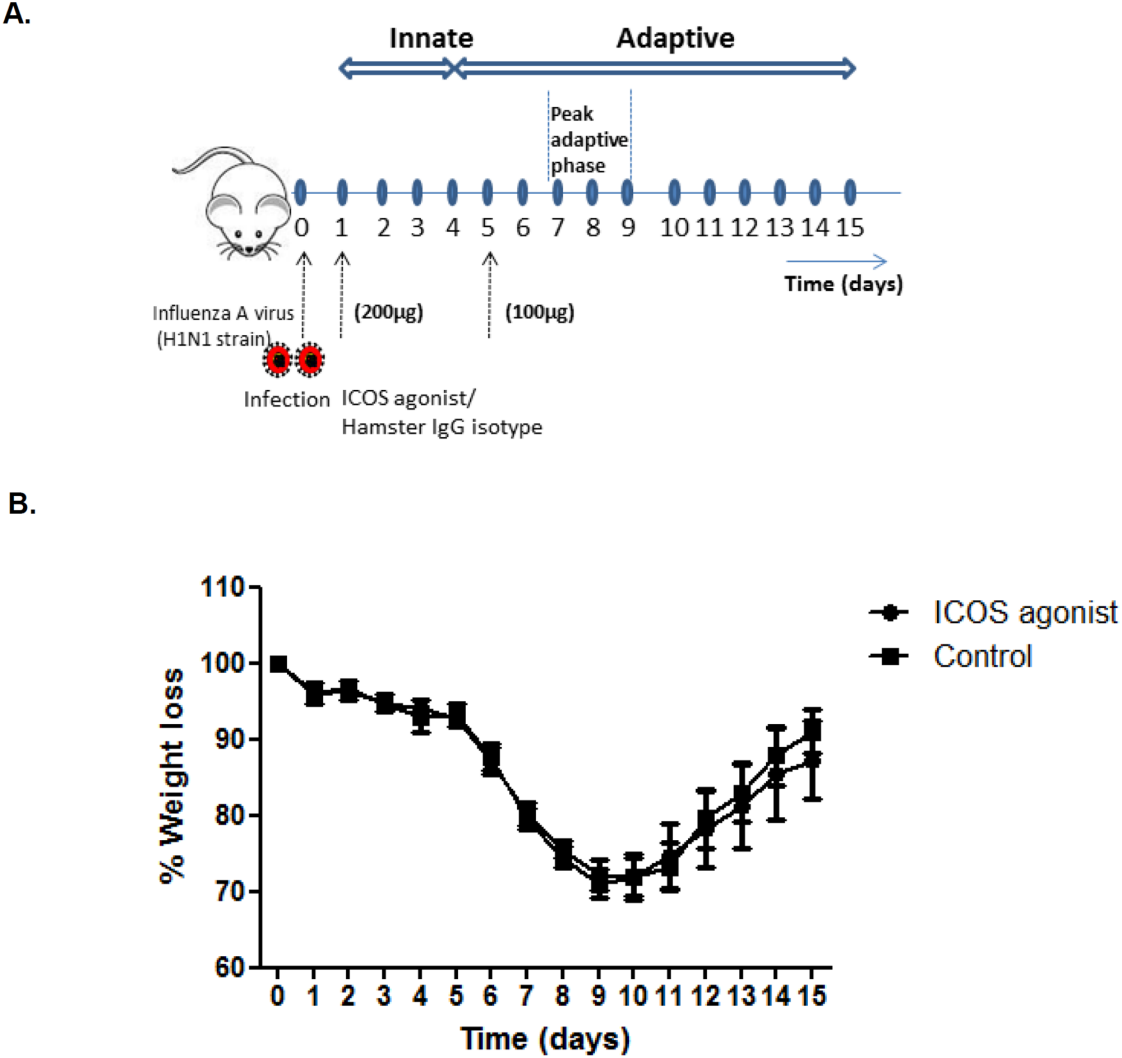
Schematic representation of the experimental procedure. (A) BALB/c mice were infected intranasal with a sub-lethal dose of IAV on day 0. Intraperitoneal injection of ICOS agonistic antibody or hamster IgG isotype control antibody was performed on day 1 (200 µg/mouse) and day 5 (100 µg/mouse) post infection followed by body weight monitoring for 2 weeks. For functional analyses, mice were sacrificed and analyzed between day 7 and 9 post infection, i.e. during the peak of adaptive immunity. (B) Mice were IAV infected and treated with ICOS agonist (n = 5) or PBS (n = 5) as described above and body weight was monitored over a time period of two weeks post IAV infection (non-parametric Mann-Whitney test was used to observe weight difference between two groups).

To study the impact of *in vivo* ICOS agonist treatment on the course of influenza infection, we used the experimental set-up summarized in **Figure 1A**. Female BALB/c mice were infected intranasal with a sub-lethal dose of influenza virus (IAV) followed by systemic ICOS agonist treatment. FACS analysis revealed rapid induction of ICOS expression on CD4^+^ and CD8^+^ T cells early after influenza infection (**Figure S3**). Taking further into account a limited half-life of the ICOS agonist antibody *in vivo* we performed ICOS agonist treatment on day 1 and 5 post infection to ensure its effect on T cells during the early and late phase of infection. Body weight loss as an indicator for disease severity was monitored, over the depicted time period of 2 weeks (**Figure 1B**). Apart from a mild delay in weight gain in the ICOS agonist treated group during the recovery phase, no significant differences in body weight loss was observed between the groups indicating that ICOS agonist treatment during the acute phase of IAV infection does not affect the overall course of the infectious disease.

### ICOS agonist treatment reduces the CD8^+^ T cells frequency in respiratory tract of IAV infected mice

**Figure 2:**
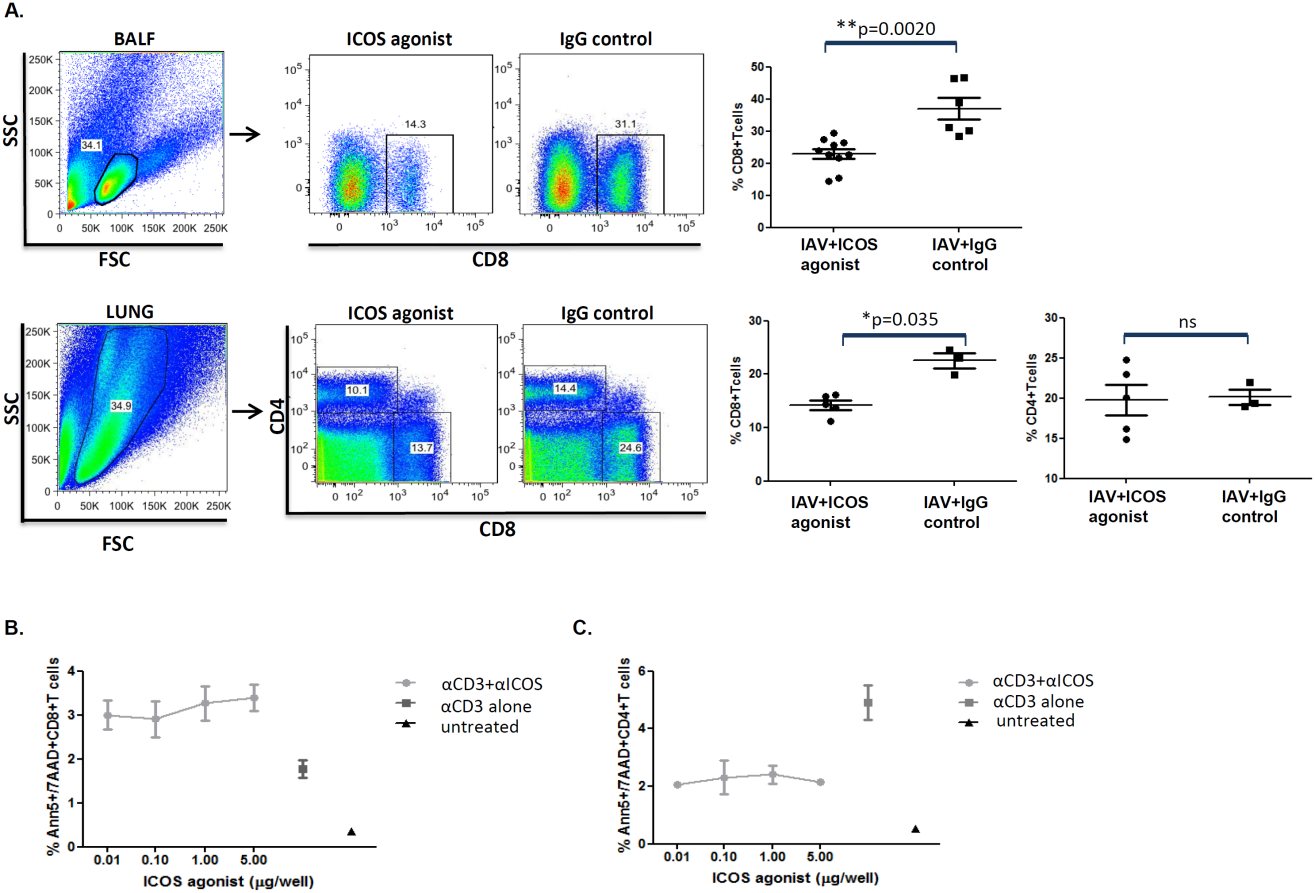
ICOS agonist treatment reduces the frequency of CD8^+^ T cells but does not affect the polyclonal CD4^+^ T cell compartment. IAV infected BALB/c mice were treated with ICOS agonist or hamster IgG isotype (control antibodies) as depicted in Figure 1A. On day 7–9 post infection the percentage of CD8^+^ in BAL (day 7, pooled data from two independent experiments) and lung (day 8) as well as CD4^+^ T cells in lung (day 9) was determined by flow cytometry. The dots in all experiments represent data from individual mice (A). *In vitro* apoptosis assays were performed, as described in materials and methods, followed by annexin-V staining on live T cells and subsequent flow cytometric analysis. Dot plot graphs indicate percentages of apoptotic CD8^+^ and CD4^+^ T cells (mean of triplicate wells) plotted against increasing concentration of ICOS agonist antibody added to the culture. CD3 alone (mean of triplicate wells) represents that cells were stimulated by anti-CD3 treatment, in the absence of ICOS agonist; untreated (mean of duplicate wells) represents that cells were neither anti-CD3 stimulated nor treated with ICOS agonist. Column statistics were performed for the apoptosis assay (the data is expressed by mean/SEM) (B and C).

Since ICOS is rapidly induced on activated CD4^+^ and CD8^+^ T cells following IAV infection (**Figure S3** and [36]), thereby making them targets for ICOS agonist binding, we first analyzed whether ICOS agonist treatment of IAV infected mice would affect the composition of the polyclonal T cell pool. Indeed, FACS analysis during the peak phase of adaptive immunity revealed a significantly reduced percentage of CD8^+^ T cells in the lung parenchyma and bronchoalveolar space (**Figure 2A**), but not in the BLN and spleen (data not shown), of ICOS agonist treated mice compared to the isotype control treated group. Moreover, the frequency of effector CD8^+^CD43^+^IFN-γ^+^ T effector cells in BAL and spleen was slightly but not significantly decreased on day 7 and 9 post infection (data not shown). Interestingly, ICOS agonist treatment had no effect on the polyclonal CD4*^+^* T cell pool (**Figure 2A** and data not shown).

To determine whether reduced pulmonary CD8^+^ T cell levels in ICOS agonist treated animals may be attributed to an enhanced apoptosis induction in activated T cells, we performed *in vitro* apoptosis assays. For this purpose, splenic T cells were stimulated with α-CD3 and cultivated in the presence or absence of ICOS agonist. Indeed, annexin V staining revealed an increased percentage of apoptotic CD8^+^ T cells in ICOS agonist treated cells compared to CD8^+^ T cells stimulated in the absence of ICOS agonist (**Figure 2B**). The opposite was found for CD4^+^ T cells which appeared to even be protected from apoptosis induction following TCR stimulation in the presence of ICOS agonist (**Figure 2C**). Together, these data demonstrate that ICOS agonist treatment during acute IAV infection reduces CD8^+^ T cell levels in the respiratory tract, which may be, at least in part, due to the induction of apoptosis in activated CD8^+^ T cells.

### ICOS agonist treatment results in delayed pathogen elimination from the infected lung

**Figure 3:**
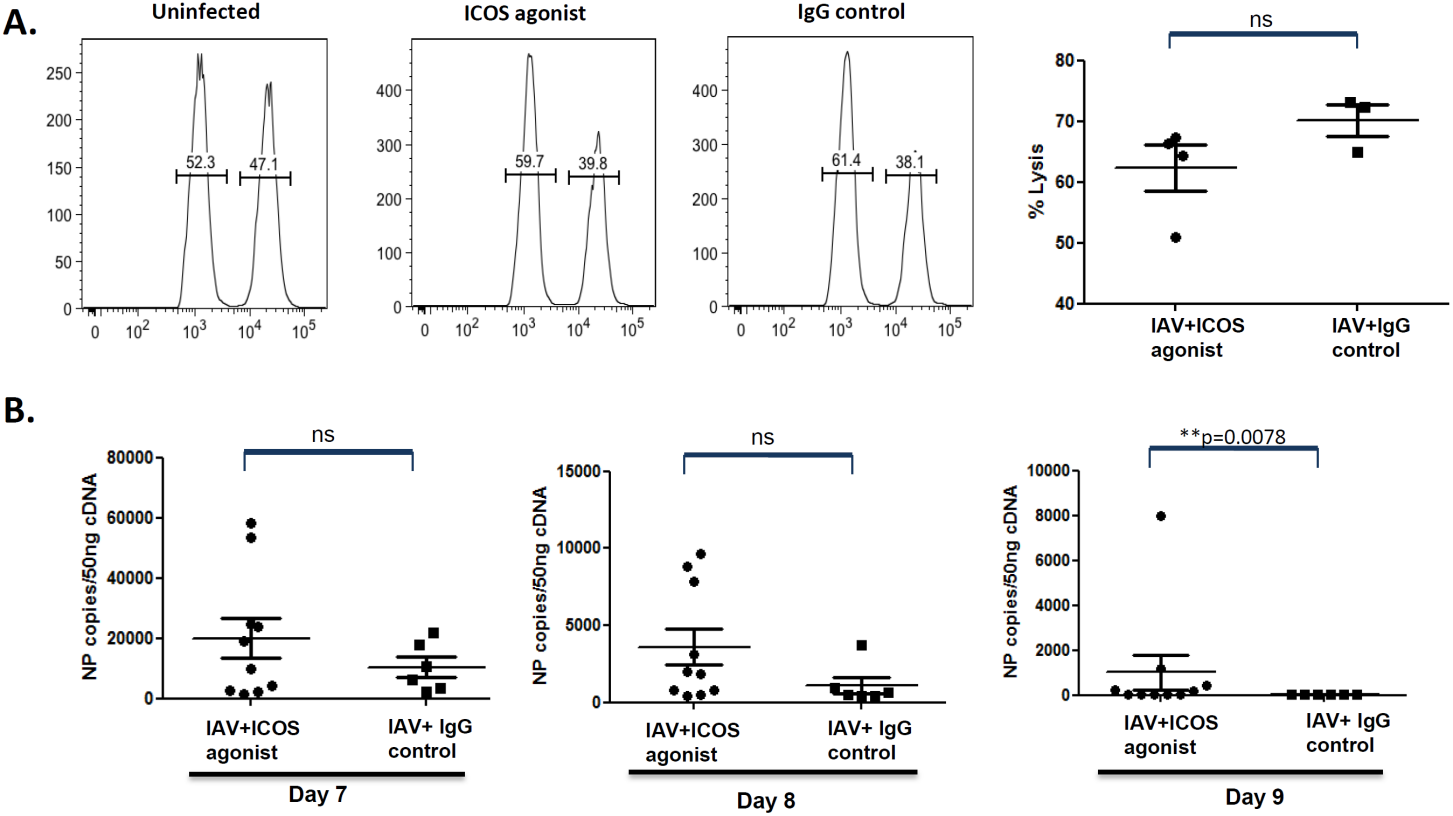
Delayed virus clearance as a consequence of ICOS agonist treatment during acute IAV infection in mice. (A) BALB/c mice were infected and treated with ICOS agonist or control antibody as depicted in Figure 1A. On day 9 post infection, influenza-specific *in vivo* CTL assays were performed, as described in materials and methods. Dot plots represent percentage specific lysis of peptide-loaded APCs by IAV-specific cytotoxic T cells in ICOS agonist and isotype control treated mice. Every dot represents data obtained for an individual mouse. (B) BALB/c mice were treated as described before with ICOS agonist antibodies and the respective isotype control antibodies. On day 7, 8 and 9 post IAV infection mice were sacrificed and NP copy numbers as an indicator for viral load was determined in the lung by quantitative RT-PCR. Dots represent data obtained for individual mice. The data were pooled from two independent experiments. Statistical analysis was done by non-parametric Mann-Whitney test.

To assess whether the ICOS agonist-mediated reduction in CD8^+^ T effector cells would result in impaired IAV-specific cytotoxicity, *in vivo* CTL assays were performed based on the adoptive transfer of IAV nucleoprotein (NP) peptide loaded splenocytes into ICOS agonist treated and control mice on day 9 post infection. Indeed, we observed a slightly reduced lysis of peptide-pulsed APC in the ICOS agonist treated group, although this did not reach the level of statistical significance (**Figure 3A**). Since viral clearance is largely dependent on CTL activity, we next analyzed whether reduced CD8^+^ T effector cell levels and cytotoxic activity would affect pathogen elimination in the lung. For this purpose, viral load was determined on day 7, 8, and 9 post infection. Whereas control animals had cleared the virus by day 9 post infection, ICOS agonist treated mice displayed higher virus titers at all times analyzed and showed a delay in virus elimination (**Figure 3B**). Notably, delayed viral clearance in ICOS agonist treated mice was as well evident when compared to mice treated with ICOS antagonist antibody, which transiently blocks ICOS signaling in T cells (data not shown), further corroborating that the observed effect can be attributed to the agonistic action of ICOS.

### ICOS agonist treatment attenuates immune-mediated influenza pneumonia

**Figure 4:**
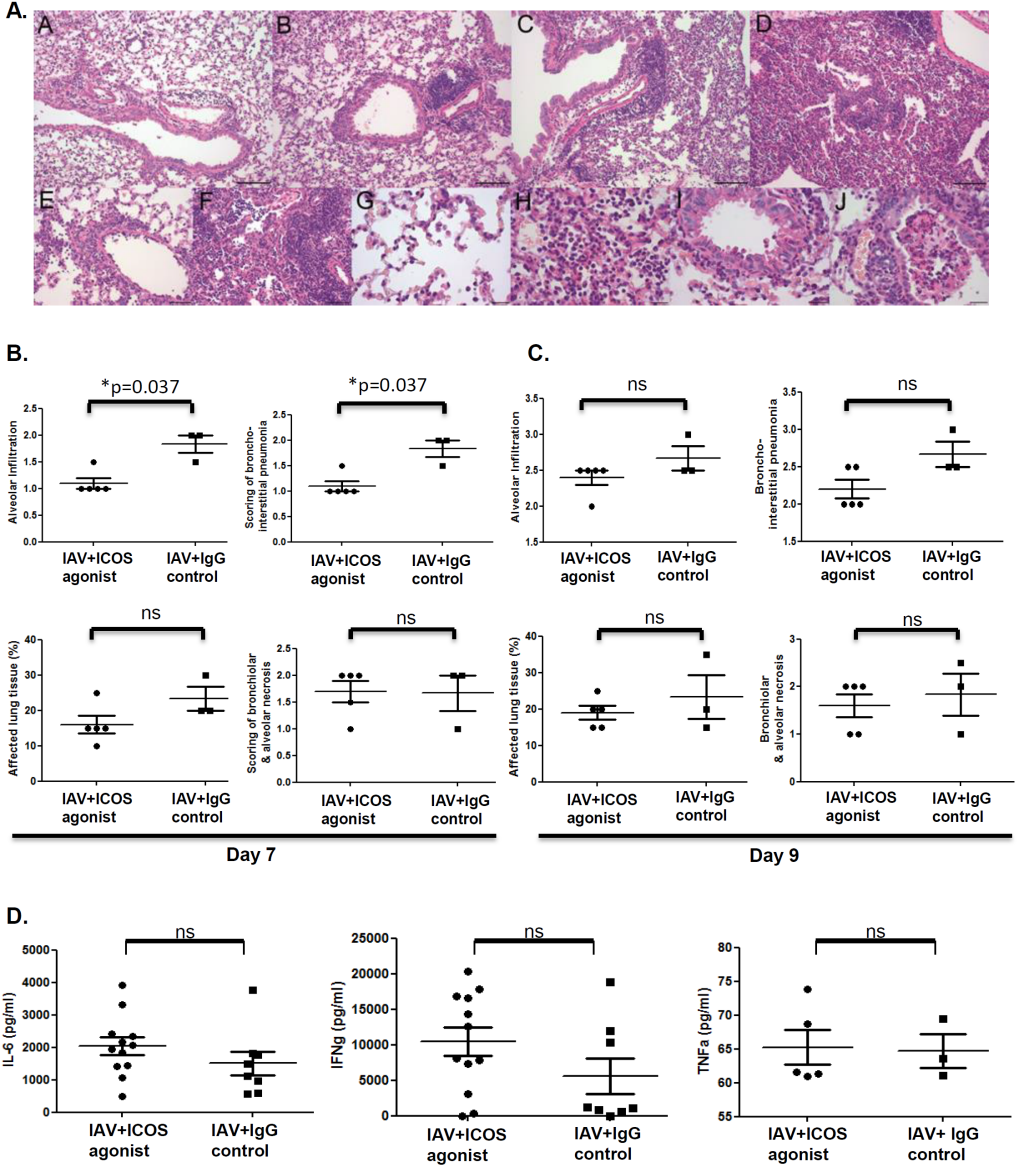
Attenuated immune-mediated pneumonia in ICOS agonist treated mice. BALB/c mice were IAV infected and treated with ICOS agonist and isotype control antibody as depicted in Figure 1A. On day 7 and 9 post infection lungs were collected and subjected to histological examination. (A) A – ICOS agonist, day 7: bronchiointerstitial pneumonia, grade 1, lymphocytic and neutrophilic; alveolar infiltration, grade 1, with lymphocytes, neutrophils and fewer alveolar histiocytes; B – isotype control, day 7: bronchiointerstitial pneumonia, grade 2, lymphocytic, with scattered neutrophils; alveolar infiltration, grade 1, with lymphocytes, fewer neutrophils and alveolar histiocytes; C – ICOS agonist, day 9: bronchiointerstitial pneumonia, grade 2, lymphocytic, with scattered neutrophils; alveolar infiltration, grade 2, with lymphocytes, fewer neutrophils and alveolar histiocytes; D – isotype control, day 9: bronchiointerstitial pneumonia, grade 2.5, lymphocytic, with scattered neutrophils; alveolar infiltration grade 2.5, with lymphocytes, fewer neutrophils and alveolar histiocytes; bronchial epithelial necrosis, grade 2; E – ICOS agonist, day 7: bronchointerstitial pneumonia, grade 1; F – isotype control, day 7: bronchointerstitial pneumonia, grade 3; G- ICOS agonist, day 7: Alveolar infiltration, grade 1; H - isotype control, day 7: Alveolar infiltration, grade 3; I - ICOS agonist, day 7: Necrosis of bronchial epithelium, grade 1; J – isotype control, day 7: Necrosis of bronchial epithelium: grade 2. (B and C) Inflammation score for alveolar infiltration, broncho-interstitial pneumonia, affected lung tissue and bronchiola & alveolar necrosis on day 7 and 9, respectively, post IAV infection. 1 = mild; 1.5 = mild to moderate; 2 = moderate; 2.5 = moderate to severe; 3 = severe; % = area of affected tissue; n = 5 mice in the ICOS agonist treated group, n = 3 mice in the isotype treated group. (D) Bronchoalveolar lavage fluid was collected on day 8 post IAV infection and concentration of IL-6, IFN-γ (data obtained were pooled from two independent experiments) and TNF-α was determined by ELISA. Dots represent data obtained for individual mice. Statistical analysis was done by non-parametric Mann-Whitney test.

We next investigated whether ICOS agonist treatment would have an effect on the severity of lung inflammation. Histological analysis performed on day 7 and 9 post infection revealed the typical signs of influenza pneumonia with multifocal, lymphocytic, bronchointerstitial pneumonia and alveolar infiltration with mainly lymphocytes, fewer neutrophils and macrophages in both experimental groups (**Figure 4A**). However, in ICOS agonist treated animals, immune-mediated inflammation was ameliorated as indicated by significantly reduced bronchointerstitial and alveolar infiltrations, as well as a decrease in the extent of lung tissue affected by the inflammation in comparison to control mice on day 7 post infection. In contrast, no differences were observed for virus-related epithelial necrosis (**Figure 4B**). Moreover, we did not observe any histological differences between the groups on day 9 post infection (**Figure 4C**). Next to histological evaluations of the lung tissue, we quantified pro-inflammatory cytokine levels in bronchoalveolar lavage fluid (BALF) collected from ICOS agonist treated and control mice. As depicted in (**Figure 4D**) BALF concentrations of IL-6, IFN-γ and TNF-α in IAV infected animals were not affected by ICOS agonist treatment. In conclusion, histopathological scoring revealed a positive effect of ICOS agonist treatment on immune-mediated pneumonia and this beneficial effect is only evident at day 7 but not at day 9 post infection.

### ICOS agonist treatment increases the frequency of CD4^+^Foxp3^+^T_regs_ and BALF levels of IL-10 cytokine

**Figure 5:**
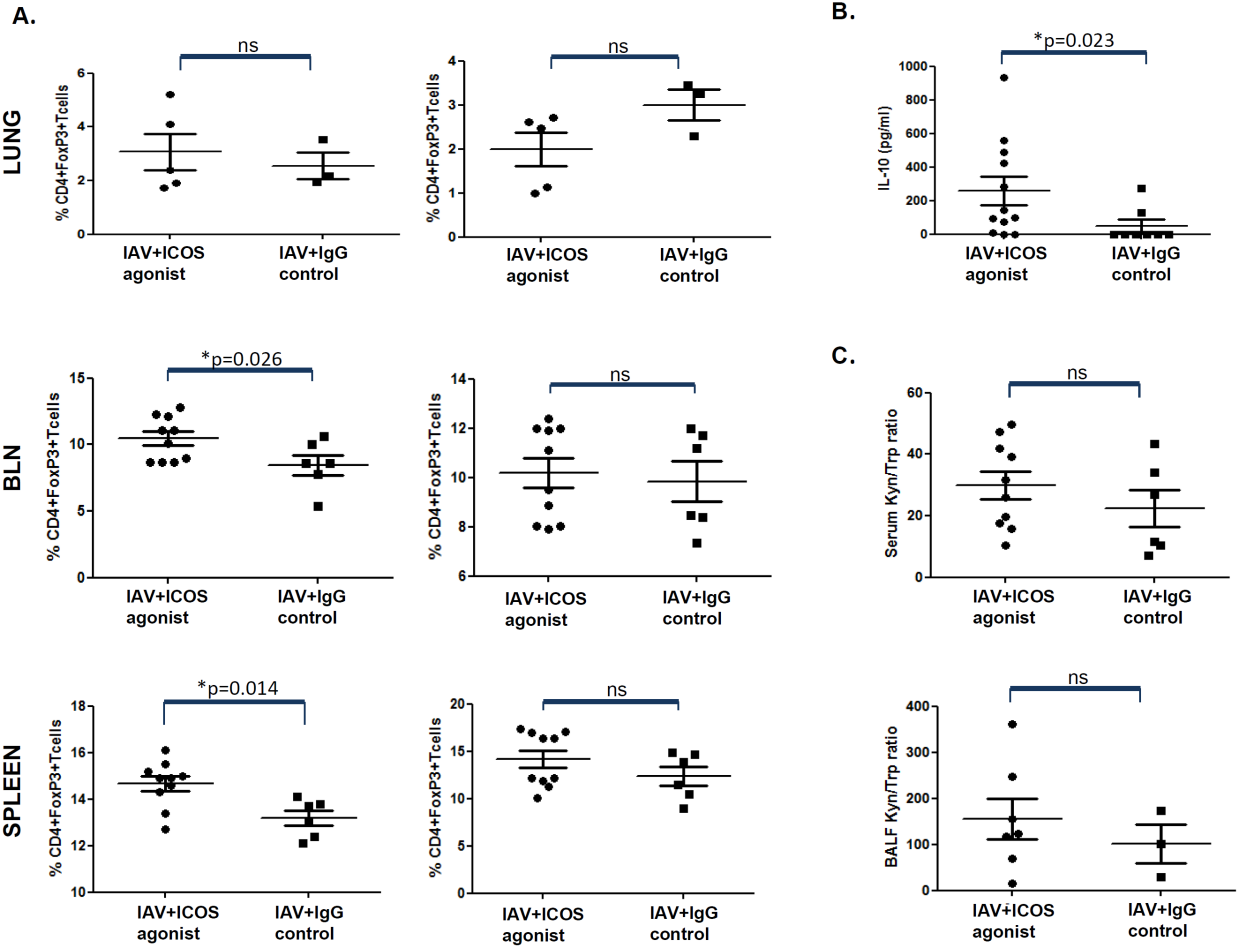
Increased frequency of T_regs_ and elevated IL-10 levels in mice treated with ICOS agonist during acute IAV infection. (A) Mice were treated as described in Figure 1A. On day 7, 8 and 9 post infection, mice were sacrificed and CD4^+^ T cells isolated from the lung (left panel: day 8 p.i.; right panel: day 9 p.i.), BLN (left panel: day 7 p.i.; right panel: day 9 p.i.) (pooled data from two independent experiments) and spleen (left panel: day 8 p.i.; right panel day 9 p.i.) (pooled data from two independent experiments) were analyzed for the intracellular expression of the T_reg_-specific transcription factor Foxp3. (B) Bronchoalveolar lavage fluid of mice infected with IAV and treated with ICOS agonist or control antibody was collected on day 8 post infection and IL-10 concentration was determined by ELISA. The data obtained were pooled from two independent experiments (C) In addition, tryptophan (trp) and kynurenine (kyn) concentrations were determined in serum samples (pooled data from two independent experiments) and bronchoalveolar lavage fluid by reversed-phase HPLC technology. IDO enzymatic activity is estimated as kyn/trp ratio. Dots represent data obtained for individual mice. Statistical analysis was done by non-parametric Mann-Whitney test.

Next to being induced on activated T cells, ICOS is also expressed on CD4^+^ T_regs_ which are known to play a key role in balancing aggressive immune responses, thereby preventing overwhelming immunopathology in infected tissues. To test whether attenuation of lung inflammation would be accompanied with the expansion of T_regs_ upon ICOS agonist treatment, the frequency of CD4^+^Foxp3^+^ T_regs_ during the peak phase of adaptive immunity was compared in mice treated with ICOS agonist or control antibody. Interestingly, we indeed found significantly increased percentages of T_regs_ in BLN and spleens, as well as slightly augmented T_reg_ levels in the lung of ICOS agonist treated animals on day 7 or 8, but not on day 9 post infection (**Figure 5A**). In line with this, we observed significantly increased concentrations of the anti-inflammatory cytokine IL-10 in the bronchoalveolar space of ICOS agonist treated mice on day 8 post infection (**Figure 5B**) as well as slightly elevated BALF and serum kyn/trp which indicates accelerated tryptophan breakdown that is most probably due to activity of the immune regulatory enzyme indoleamine 2,3-dioxygenase (IDO) (**Figure 5C**). Together, our data suggest a beneficial effect of ICOS agonist treatment during acute IAV infection on the severity of lung inflammation by decreasing the frequency of CD8^+^ T cells but at the same time supporting the expansion of T_regs_.

## Discussion and Conclusion

While previous studies on the specific function of ICOS signaling in inflammatory and infectious conditions have been performed in mice lacking ICOS or by using antibody-mediated blockade of ICOS function, to our knowledge this is the first report on the utilization of a stimulating ICOS antibody for *in-vivo* immune modulation. Based on results obtained from previous infection studies in the absence of ICOS [5], [14], [15], [27]–[31], we hypothesized that a specific stimulation of ICOS during an acute IAV infection should affect the induction and/or regulation of virus-specific T cell responses with potential implications for pathogen elimination and immune-mediated pathology.

We first characterized the overall impact of ICOS agonist on the composition of the polyclonal T cell pool during the peak phase of adaptive immunity. While no effect of ICOS agonist treatment was observed for the frequency of respiratory CD4^+^ T cells, we found significantly decreased levels of CD8^+^ T cells in the lung and BALF as well as slightly reduced frequencies of CD43^+^IFN-γ^+^ CD8^+^ T_eff_ (**Figure 2** and data not shown). This was somehow unexpected since ICOS/ICOS-L interaction has been demonstrated before in a tumor model to improve priming of naïve CD8^+^ T cells [38], [39]. Moreover, a drop in CD8^+^ (and CD4^+^) T cells was also observed in IAV infected mice treated with an antibody blocking ICOS [30]. Another study using IAV infection in ICOS^−/−^ mice revealed no differences in primary expansion of virus-specific CD8^+^ T cells [32]. Very importantly, in contrast to these studies focusing on CD8^+^ T cell expansion in IAV infected mice in the absence of functional ICOS signaling, we super-stimulated ICOS signaling. Reduced CD8^+^ T cell levels in ICOS agonist treated animals may be due to the existence of a negative feedback loop controlling excessive division and differentiation of primed CD8^+^ T cell or may be due to increased cell death specifically induced in CD8^+^ T cells. The former concept is supported by the observation that ICOS stimulation during ongoing infection supports the expansion of T_regs_ and the establishment of an immunosuppressive environment. The latter concept is supported by our data obtained in *in-vitro* apoptosis assays revealing that T cell stimulation in the presence of ICOS agonist predisposes CD8^+^ (but not CD4^+^) T cells for death by apoptosis (**Figure 2**). This is well in line with another study that demonstrated differential effects of ICOS on CD4^+^ and CD8^+^ T cells with an increased ability of ICOS^−/−^ CD8^+^ T cells to induce Graft-versus-Host Disease (GvHD) as a result of enhanced survival and expansion of those cells [40].

Since CD8^+^ T cells are pivotal for the efficient elimination of IAV from the lung [41], reduced CD8^+^ T cell levels should give rise to failures in antiviral immunity and pathogen clearance. This was indeed what we observed in our study. An earlier study reported [32] that ICOS deficient mice show normal IAV-specific CTL responses compared to mice sufficient for ICOS signaling, ICOS agonist treated animals showed a slightly reduced tendency for IAV-specific cytotoxic T cell function (**Figure 3**), which correlated with delayed pathogen elimination from the respiratory tract. Whereas control animals cleared the virus within 9 days post infection, virus was still detectable in the lungs of mice treated with agonistic ICOS antibody at this time (**Figure 3**). Reduced CD8^+^ T cell frequencies and CTL activity together with delayed pathogen elimination are very well in line with the finding that ICOS agonist treated animals exhibited a less pronounced immunopathology in the lung. Importantly, this applied exclusively to histological parameters indicating immune-mediated inflammation such as bronchointerstitial pneumonia, alveolar infiltrations and the extent of lung tissue affected by the inflammation (**Figure 4**), whereas no impact of ICOS agonist treatment was detectable for bronchiolar and alveolar necrosis which is largely induced by the pathogen itself.

Our finding that ICOS agonist treatment during the acute phase of IAV infection improves immune-mediated pathology prompted us to further investigate whether super-stimulation of the ICOS signaling pathway would favor the induction of immune regulatory mechanisms. Interestingly, we indeed observed a significant increase in the frequency of T_reg_ in BLN and spleen on day 7 and 8 post infection, while the T_reg_ level in the lung of ICOS agonist treated mice was only slightly increased. Expansion of T_reg_ by targeted activation of the ICOS signaling pathway is very well in line with previous reports demonstrating that ICOS is required for the development of T_reg_ [26], [31]. Very recently, it has been shown that ICOS co-stimulation promotes the expansion and maintenance of Foxp3^+^ T_reg_ during helminthes infections [42]. BALF concentration of the anti-inflammatory cytokine IL-10 was increased in the ICOS agonist group which is consistent with reports showing the super-synthesis of IL-10 by ICOS-expressing T_regs_ [43]. Based on published data [44] we hypothesized that IFN-γ induced in activated T cells in the context of ICOS signaling would act in a paracrine manner on adjacent APCs resulting in the production of IDO [45]. However, analysis of serum and BALF activity of the immune regulatory enzyme IDO revealed only marginal differences between the two experimental groups with slightly higher IDO activity detectable in mice that were treated with the ICOS agonist. The trend was similar to that observed for IFN-γ and IL-6 levels, but did, however, not reveal significant differences. IL-10 and IDO are well known to create a regulatory milieu and both mediators have been described to inhibit the proliferation and differentiation of effector T cells [46]–[48]. It may be speculated that an increased apoptosis induction in CD8^+^ T cells following priming in the presence of ICOS agonist, together with elevated concentrations of immunosuppressive mediators, may account for the observed decline in CD8^+^ T cells in the respiratory tract of IAV infected animals. Another important fact to be considered in this context is that elevated T_reg_ numbers in the lymph nodes draining the site of acute virus infections and in the spleen may negatively affect CD8^+^ T cell priming [49], [50]. Although, partial depletion of CD25^+^ T_reg_ has been recently shown to have no effect on IAV induced mortality, weight loss, viral clearance and cellularity within the lung [51], we speculate that increased T_reg_ levels in the BLN and spleen of ICOS agonist treated mice may interfere with efficient priming of IAV-specific CD8^+^ T cells. Inefficient CD8^+^ T cell priming combined with an immunosuppressive milieu in the lung that has a negative effect on the proliferation and effector cell differentiation would result in reduced CD8 T_eff_ cells with beneficial outcome for immune-mediated influenza pneumonia. This is exactly what we observe in mice treated with ICOS agonist during acute IAV infection.

Previous investigations on the impact of ICOS signaling on the course of infection and pathogen clearance had opposing outcome dependent on the infection model used. Studies on the role of ICOS in the control of systemic *Salmonella typhimurium* carried out in ICOS^−/−^ mice supported the importance of ICOS for immunoglobulin class switch and efficient induction of pathogen-specific CD8^+^ T cell responses. In line with this, ICOS deficiency resulted in delayed bacterial clearance [15] thus suggesting a critical role of ICOS in the control of Salmonella infection. Another study performed in ICOS^−/−^ mice addressed the impact of ICOS on T cell responses and protection against *Mycobacterium tuberculosis* (Mtb). In direct contrast to the Salmonella model, ICOS deficient mice showed reduced Mtb bacterial burden in the chronic phase of infection in the spleen (but not the lung) while immunopathology was unaltered between knock out and wild type mice [31]. Here, intriguingly and in support of our data, the authors found that ICOS deficiency differentially affected CD4^+^ and CD8^+^ T cell subsets. In their model, the polyclonal CD4^+^ T cell response against Mtb was improved which came along with diminished frequencies of T_regs_. Conclusively, while we demonstrate the expansion of T_reg_ following stimulation of the ICOS signaling pathway in acute IAV infection, the opposite was found in Mtb infection in mice lacking ICOS signaling. Interestingly, Mtb infection in ICOS^−/−^ mice resulted in reduced numbers of pathogen-specific CD8^+^ T_eff_ cells and reduced CTL activity in the chronic phase of infection [31], which was demonstrated also by us when we stimulated ICOS signaling. Possible explanations for this obvious discrepancy may be due to the fundamental differences in the infection models used (viral *versus* bacterial, acute *versus* chronic, local *versus* systemic) and that we analyzed the T cells much earlier, i.e. already during the peak phase of adaptive immunity. Improved Mtb clearance despite reduced CTL activity might most probably account for the fact that unlike in IAV infection, Mtb clearance is predominantly dependent on CD4^+^ T cells [52]. Next to bacterial infections, the impact of ICOS signaling has also been studied for viral pathogens including VSV, LCMV and IAV infection. Depending on the viral pathogen analyzed, these studies also revealed an important impact of ICOS signaling for the different arms of adaptive immunity [32], [53]. Importantly, antibody-mediated inhibition of ICOS during IAV infection resulted in reduced CD8^+^ T cell numbers, pulmonary T cell-inflammation and impaired control of IAV replication in the lung [30], thereby largely resembling the findings from our study. While the overall outcome of ICOS inhibition and ICOS stimulation during acute IAV infection appears to be similar, there may be considerable differences in the underlying immunological mechanisms. Whereas we found that ICOS super-activation predisposes CD8^+^ T cells to apoptosis, ICOS inhibition did not enhance T cell apoptosis in the lung. While our data suggest that ICOS stimulation expands the T_reg_ pool and that elevated T_reg_ levels in combination with increased IL-10 concentrations in the respiratory tract inhibits CD8^+^ T cell priming and effector cell differentiation, T_reg_ were not analyzed in the study by Humphreys and colleagues. Taken together, these mouse infection studies clearly indicate that ICOS acts as important modulator of adaptive immune responses and balances CD4^+^ T_helper_, T_reg_ and CD8^+^ T_eff_ cell responses, thereby representing a potential target for the modulation of pathogen-specific immunity.

In conclusion, to our knowledge, this is the first *in-vivo* study addressing the specific impact of ICOS on the outcome of infection utilizing ICOS activation rather than blocking. Super-activation of ICOS during acute IAV infection was found to modulate the respiratory and peripheral T cell pool resulting in an increased T_reg_/CD8^+^T cell ratio and delayed pathogen elimination from the lung. Attenuated antiviral immunity however ameliorated immune-mediated influenza pneumonia making ICOS an interesting target for therapeutic intervention in those infectious conditions characterized by strong immuno-pathology rather than virus-mediated cytopathic effects.

## Supporting Information

Figure S1 **Minor blockade of ICOS agonist binding to activated T cells by soluble ICOS-L pre-incubation.** Splenic T cells were pre-activated *in vitro* with PHA and IL-2 for 72 hours followed by the addition of soluble ICOS-L B7h-Fc. Control cells were not treated with B7h-Fc. After 30 minutes incubation T cells were stained with the ICOS agonist antibody C398.4A-FITC and binding of ICOS agonist to T cells in the presence or absence of ICOS-L was analyzed by FACS. Left panel shows representative histograms; right panel summarizes data as mean fluorescence ratio (mean ± SE) obtained from three individual experiments.
(TIF)

Figure S2 ***In vivo*** **treatment of mice with ICOS agonist does not result in depletion of ICOS^+^ T cells.** Mice were treated with ICOS agonist (C398.4A) or hamster IgG as negative control. After 72 hours mice were sacrificed and the proportion of TCRαβ^+^ICOS^+^cells in the spleen was determined by FACS. Shown are means ± SE from three individual experiments.
(TIF)

Figure S3 **Expression of ICOS on CD4^+^ and CD8^+^ T cells at different time post influenza virus infection.** Mice were infected with a sublethal dose influenza A virus as described in materials and methods. On day 1, 5, 7, 10 and 14 post infection mice (n = 3) were sacrificed and the percentage of ICOS^+^ CD4^+^ and CD8^+^ T cells in bronchoalveaolar lavage fluid, lung, BLN and spleen was determined by FACS analysis.
(TIF)
