# Supplementary figures and images for: Attenuation of Immune-Mediated Influenza Pneumonia by Targeting the Inducible Co-Stimulator (ICOS) Molecule on T Cells
Source: PLoS One. 2014 Jul 16;9(7):e100970. doi: 10.1371/journal.pone.0100970 (PMC4100737; doi:10.1371/journal.pone.0100970)

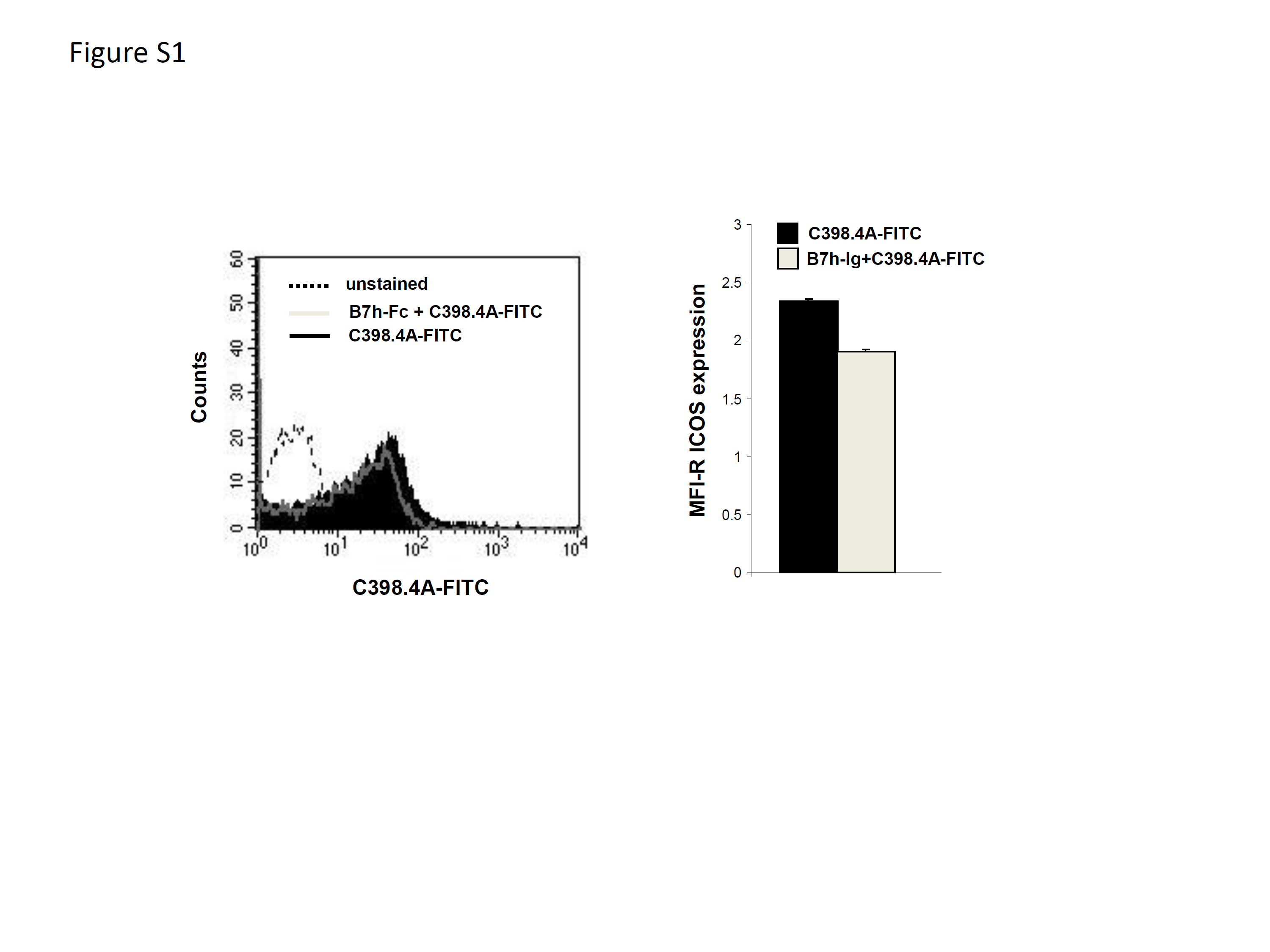

Supplement: Figure S1 — Minor blockade of ICOS agonist binding to activated T cells by soluble ICOS-L pre-incubation. Splenic T cells were pre-activated in vitro with PHA and IL-2 for 72 hours followed by the addition of soluble ICOS-L B7h-Fc. Control cells were not treated with B7h-Fc. After 30 minutes incubation T cells were stained with the ICOS agonist antibody C398.4A-FITC and binding of ICOS agonist to T cells in the presence or absence of ICOS-L was analyzed by FACS. Left panel shows representative histograms; right panel summarizes data as mean fluorescence ratio (mean ± SE) obtained from three individual experiments. (TIF) [file pone.0100970.s001.tif]

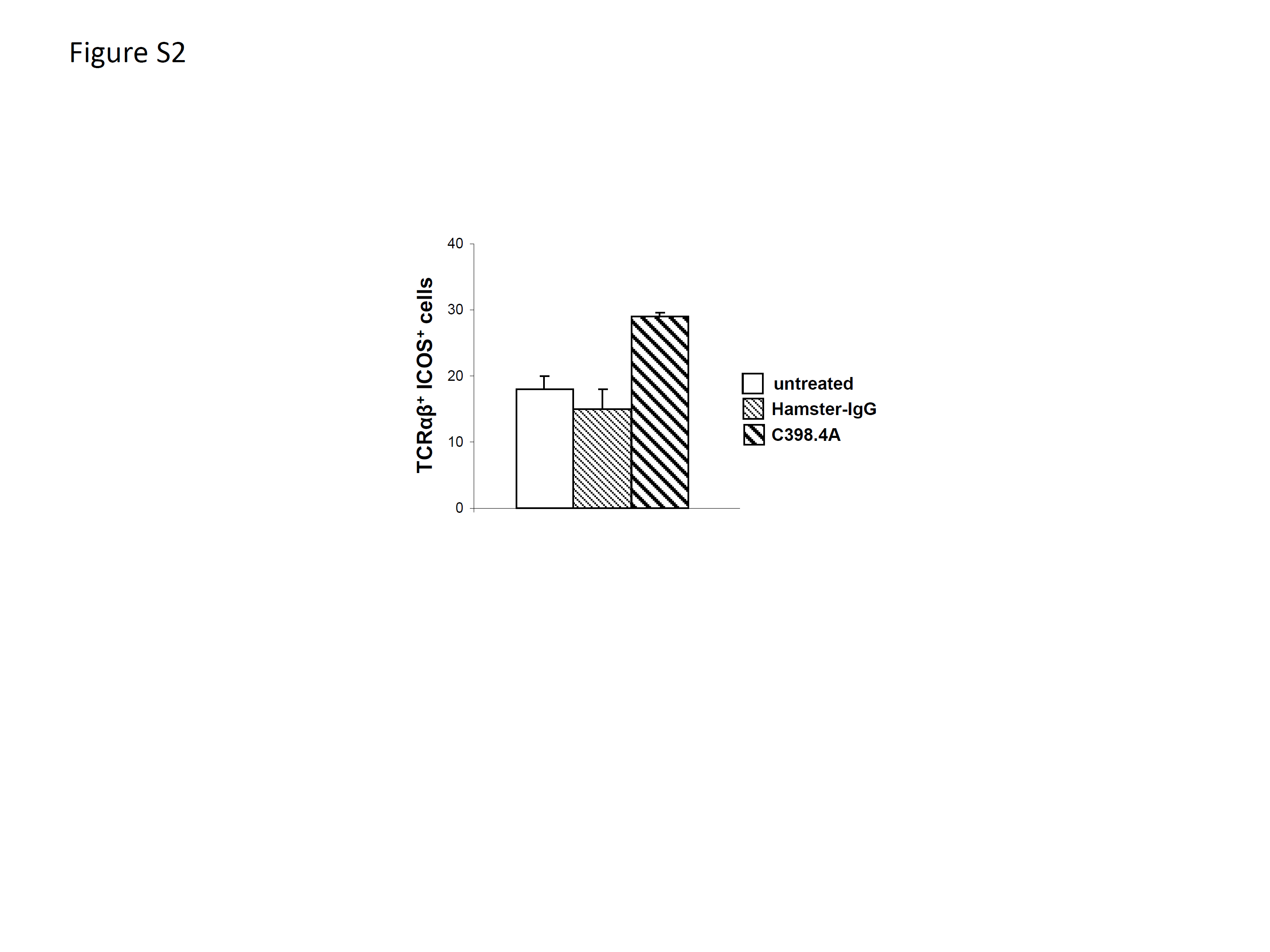

Supplement: Figure S2 — In vivo treatment of mice with ICOS agonist does not result in depletion of ICOS+ T cells. Mice were treated with ICOS agonist (C398.4A) or hamster IgG as negative control. After 72 hours mice were sacrificed and the proportion of TCRαβ+ICOS+cells in the spleen was determined by FACS. Shown are means ± SE from three individual experiments. (TIF) [file pone.0100970.s002.tif]

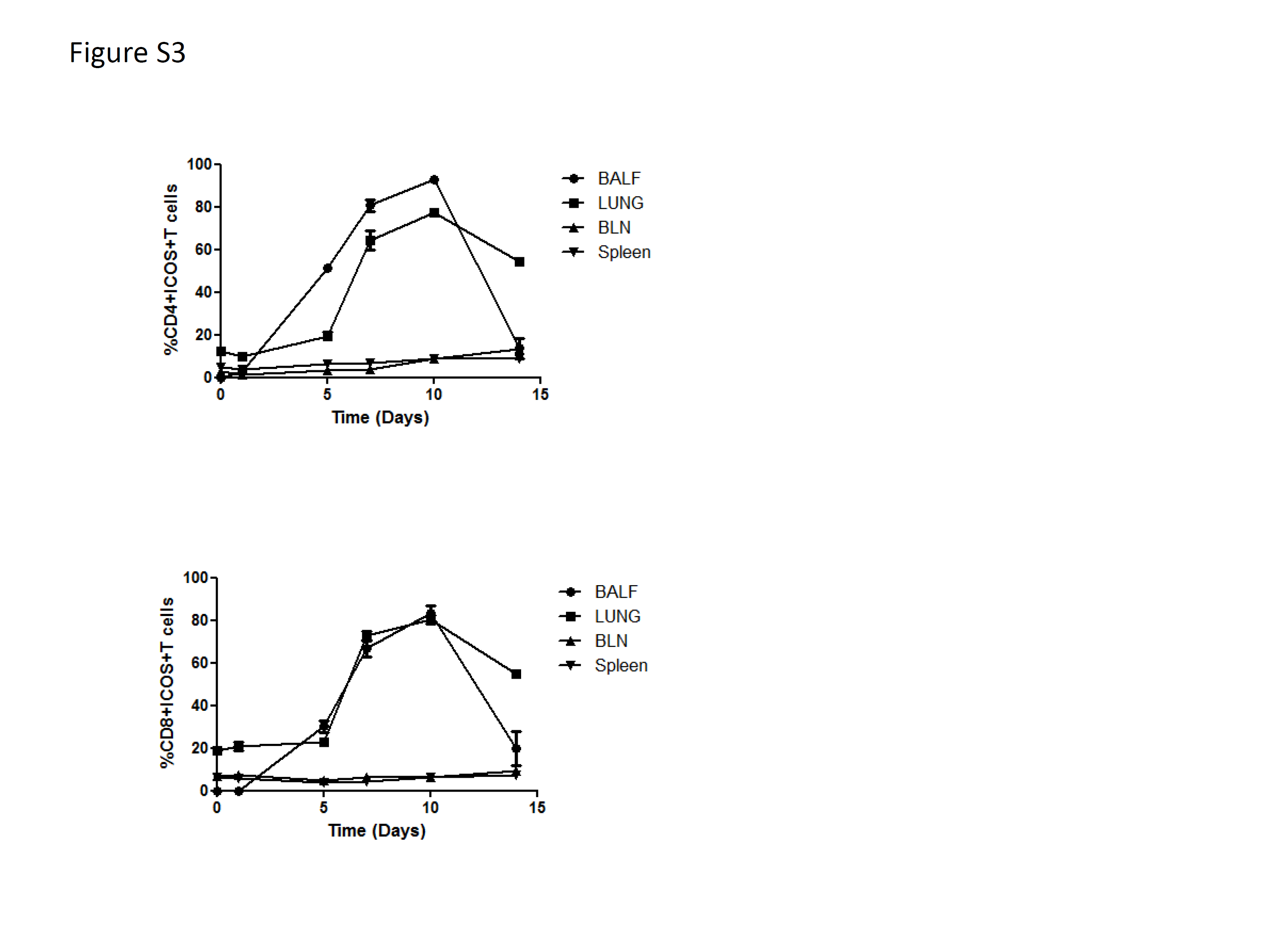

Supplement: Figure S3 — Expression of ICOS on CD4+ and CD8+ T cells at different time post influenza virus infection. Mice were infected with a sublethal dose influenza A virus as described in materials and methods. On day 1, 5, 7, 10 and 14 post infection mice (n = 3) were sacrificed and the percentage of ICOS+ CD4+ and CD8+ T cells in bronchoalveaolar lavage fluid, lung, BLN and spleen was determined by FACS analysis. (TIF) [file pone.0100970.s003.tif]
